# Assessing changes expression and presentation of NKG2D under the influence of serum factors in breast cancer patients

**DOI:** 10.1186/2051-1426-2-S1-P8

**Published:** 2014-02-24

**Authors:** Ali Khodadadi, Rayhaneh Roshani, Mehri G Boroujerdnia, Abdolhassan Talaiezadeh, Sara Iranprast

**Affiliations:** 1 Department of Immunology and Petroleum and Environmental Pollutants Research Center, Faculty of Medicine, Jundishapur University of Medical Sciences, Ahvaz, Iran; 2 Department of surgery, Petroleum and Environmental Pollutants Research Center, Faculty of Medicine, Ahvaz Jundishapur University of Medical Sciences, Ahvaz, Iran

## Introduction

Breast cancer is the most common cancer in women worldwide. NK cells play an important role in the elimination of tumor cells by NKG2D through lysis of target cells by binding to the MICA proteins. It has been demonstrated that MICA shedding from tumor cell surface. In this study was to assessing the levels of sMICA and changes expression and presentation of NKG2D on NK cells from patients PB with breast cancer.

## Method

PB was taken from 50 healthy and 50 breast cancer person before surgery and chemotherapy. NKG2D expression were assessed by qRT-PCR and analyzed using REST RG.ELISA was used to examine the sMICA in serum. The presentation of NKG2D were identified by three-color flow cytometry method. Separated PBMC Stained by Anti-CD56/anti-NKG2D/anti-CD3. Then results analyzed with WinMDI software. Statistical analysis of all data was done using SPSS software.

## Results and conclusion

Our study reveals that in contrast to healthy volunteers significant amounts of sMICA was detected in sera from nearly all patients. The expression and presentation of NKG2D was significantly lower than of healthy person. Elevated sMICA and changing in expression and presentation of NKG2D are potential markers in diagnosis stages and post treatment of breast cancer.

